# Geriatric screening in first opinion practice – results from 45 dogs

**DOI:** 10.1111/j.1748-5827.2012.01247.x

**Published:** 2012-07-27

**Authors:** M Davies

**Affiliations:** School of Veterinary Medicine and Science, University of NottinghamSutton Bonington Campus, Loughborough, LE12 5RD

## Abstract

**Objectives:**

To evaluate and report the results of screening geriatric dogs in a first opinion practice.

**Methods:**

A prospective health screen of dogs over nine-years-old involving history taking, physical examination and urinalysis.

**Results:**

At least one previously unrecognised problem was identified in 80% of 45 dogs and 353 findings (mean 7·8 per dog) were recorded. Owners often failed to recognise and report serious signs of age-related disease. However, they most often reported increased sleeping (31%), loss of hearing (29%) or sight (20%), stiffness or lameness (22%) and “slowing down” (20%). Increased lens opacity (64%), increased thirst (58%), pain (24%), increased frequency of urination (24%), signs of osteoarthritis (24%) and dental disease (22%) were most frequently identified at the time of consultation. Potentially, life-threatening findings included respiratory distress, palpable abdominal masses and metastatic lung disease. Screening resulted in 29 further diagnostic procedures, including 10 dental procedures, seven medical treatments, two surgical procedures and euthanasia of two dogs.

**Clinical Significance:**

Screening elderly dogs identified unrecognised and unreported health risk factors resulting in lifestyle modification and ongoing monitoring, as well as signs of age-related diseases resulting in diagnostic investigations, early diagnoses and surgical and medical interventions to improve quality of life.

## INTRODUCTION

The aims of screening elderly dogs are to detect occult signs of age-related disease resulting in early investigation, diagnosis and intervention, to relieve pain, to identify risk factors and problems that require ongoing monitoring and to provide management that improves quality of life, delays onset of clinical signs and, possibly, prolongs life. If no abnormal signs are found screening provides baseline information that clinicians can use as benchmarks for comparison at some later date.

Veterinarians rely on owners to observe and report signs of age-related diseases but they may not recognise cardinal signs as being important enough to report (Davies 2011), so screening is desirable and is widely recommended (Davies 1996, Hoskins 2004, AAHA 2005). Veterinary clinicians frequently perform health checks at the time of routine vaccination, but the time allocated for this is usually less than 20 minutes, and there are no publications quantifying the results of such checks. It is estimated that approximately 40% of elderly patients in a screening programme require further diagnostic tests or treatments and senior health care programmes can generate up to 35% of veterinary hospital turnover (Hoskins 2004). Guidelines for a health care programme for elderly dogs have been published (AAHA 2005); however, a search of the veterinary literature using the online databases CAB Abstracts (Ovid), Medline and PubMed (last accessed December 22, 2011) failed to find any studies reporting the results of health screening for geriatric dogs in first opinion practice.

According to the computer records of 120 UK veterinary practices (FDI 2010) 32·5 and 30% of practices ran health examinations for senior dogs or cats over seven years age, respectively. However, only 1·7% of dogs and 1·6% of cats attend those assessments in a year, even though 41% of dogs and 50% of cats registered with the practices were within the senior age group. In the same report, only 20% of animals (all ages) underwent a pre-anaesthetic haematology and serum biochemistry screen and 7·1% of senior pets had a routine urinalysis.

In a study of pre-anaesthetic screening (including haematology and serum biochemistry), new diagnoses were made in 30% of 101 dogs over seven years of age (mean 10·99 years) and 13% did not undergo anaesthesia as a result (Joubert 2007). In another study evaluating pre-anaesthetic haematology and serum biochemistry tests in 1537 dogs (mean age 5·8 years) it was concluded that blood tests were unlikely to yield additional important information if no problems were identified in the history or physical examination (Alef and others 2008) and only platelet count and alanine aminotransferase (ALT) activity showed statistically significant difference (P<0·05) in dogs aged over 10 years compared to younger dogs.

Routine blood screening is controversial as there is a high (64%) likelihood of false negative or positive results in a panel of 20 blood tests (Archer 2005a). Therefore, finding a result outside the reference limits may require further investigations to confirm its validity and costs can then become an important consideration for the owners. Diagnostic venepuncture is invasive and in humans the minor complication rate (bruising and haematoma) is 12·3% and serious complication rate (including diaphoresis with hypotension, and syncope) is 3·4% (Galena 1992). Complication rates for venepuncture in first opinion veterinary practice are not available but routine sampling is difficult to justify if the clinical value is limited (Alef and others 2008).

There is no generally accepted definition of a “geriatric dog” but the American Animal Hospital Association consider it is the last 25% of expected life span, so ages will vary as giant and large breeds have shorter life expectancy than smaller breeds (AAHA 2005).

The aim of this study was to determine whether screening geriatric dogs in a first opinion veterinary practice by taking a detailed history, performing a complete physical examination, and analysing urine with dip sticks and specific gravity measurement by refractometer would be beneficial in identifying unrecognised problems that require further investigation or ongoing monitoring.

The hypothesis was that screening geriatric dogs would result in the identification of unrecognised or unreported risk factors or signs of age-related diseases resulting in recommendations for lifestyle changes (e.g. diet), ongoing monitoring, further diagnostic investigations and medical or surgical interventions.

## MATERIALS AND METHODS

This was a prospective study conducted in a rural mixed veterinary practice. Owners with dogs aged nine years or older (arbitrarily selected for the study) were randomly identified from the practice management software in use in the practice [Megavet – Vet Solutions; the Megavet computer system (a Unix-based practice management system) is no longer available]. In batches of 100, the records were manually checked and exclusion criteria for participation in the study were if the dog had died, the owners had moved out of the area or the dog had undergone a veterinary examination at the practice within 2 months before the study.

Owners were invited by letter to attend a free 30-minute veterinary consultation consisting of a history taking session, a full -physical examination and basic urinalysis. No follow-up contact was made with the owners who did not respond to the initial mailing.

All consultations were performed by the same experienced veterinarian who did not work full time at the practice, did not know the practice clients and who was masked from the previous history of the dogs.

**FIG 1:**
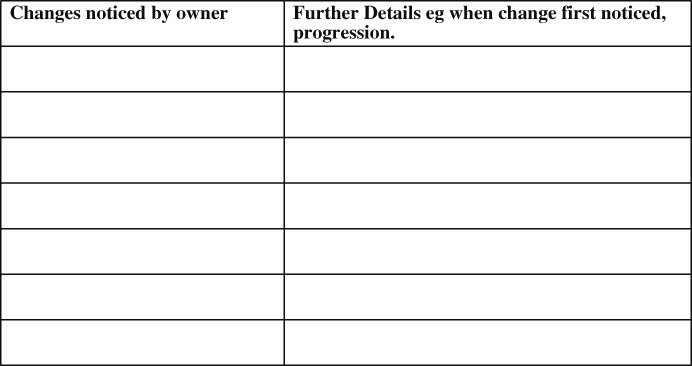
History Part 1 Subjective – changes noticed and reported by the owner without prompting “What changes have you noticed since your dog has become older?”

**FIG 2:**
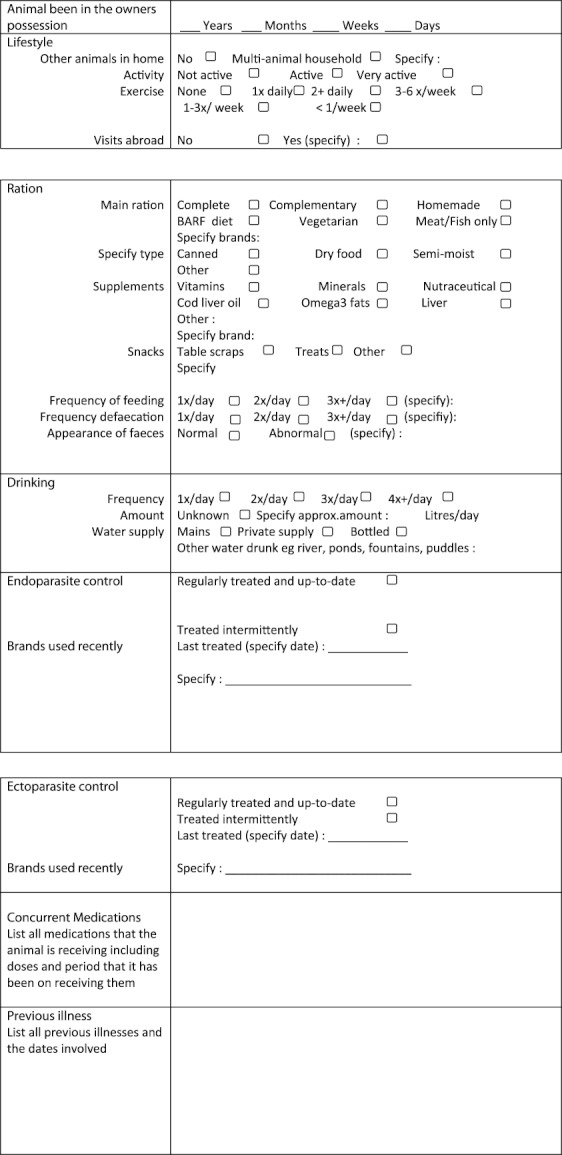
History Part 2 : Basic fact finding history

**FIG 3:**
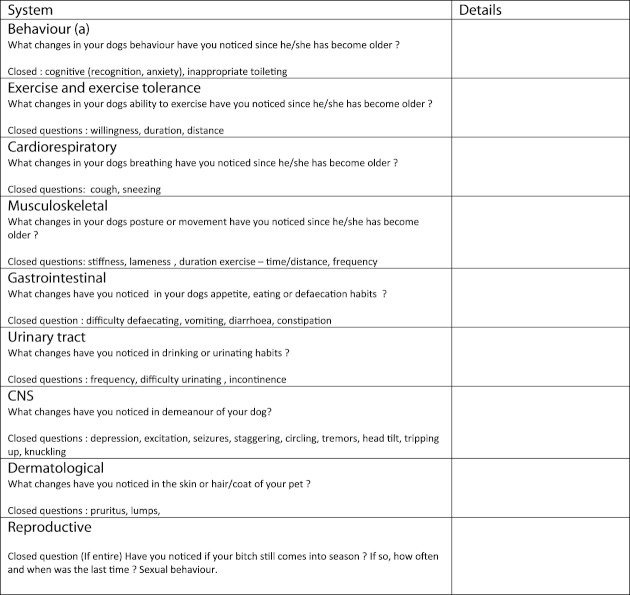
History Part 3 Subjective – history prompted by clinician questions

The history taking was standardised and divided into in three separate sections. First, the owner was asked what changes they had noticed as the dog had become older (Fig 1) and care was taken not to prompt areas of likely change such as behaviour, exercise, drinking or toileting patterns. Second, basic data collection (Fig 2) about the animal and its lifestyle was collected. Finally, a prompted history (Fig 3) was completed. Initially, open questions were asked, but before questioning on a section ended appropriate closed questions were asked.

The physical examination consisted of a full visual and hands-on examination including thoracic auscultation, ophthalmoscopy, otoscopy, abdominal palpation, digital rectal examination of entire male dogs, manipulation of limbs and axial skeleton, and neurological evaluation. A 5-point body condition score system was used.

Urinalysis included Multistix dip stick tests (Bayer) and specific gravity measured by refractometer. Urine sediment examination was not performed and blood tests were not included in the screen; however, additional diagnostic tests were recommended to the client if indicated from the history or physical examination. Clients were requested to pay for these additional tests.

## RESULTS

On average, 15 dogs were excluded per 100 retrieved from the practice management database. In total, 255 of 300 owners were invited to participate and the 18% response resulted in 45 dogs entering the study.

The time allocation of 30 minutes for the consultations proved inadequate and it was extended to a minimum of 45 minutes.

Dog ages ranged from 9 years and 4 months to 16 years (median 11 years and 6 months). Pedigree breeds accounted for 33 (73%) of the study group with 12 (27%) being crossbreeds. The dogs included 19 (45%) neutered females, 6 (13.5%) entire females, 11 (24.5%) neutered males and 9 (20%) entire males.

Changes that owners had noticed as their dog had become older are listed in Table 1.

**Table 1:** Unprompted owner observations as their dog had aged (n=45)

Observed change	Number reporting this (%)
Increased sleeping	14 (31)
Losing hearing	13 (29)
Lameness/stiffness	10 (22)
Losing vision	9 (20)
Slowing down	9 (20)
Panting a lot	5 (11)
Increased thirst	3 (7)
Urinating in house	2 (4)
Coughs when walking	1 (2)
Tires when walking	1 (2)

Only 5 (11%) of the owners in this study fed a “senior” diet. Dog treats or snacks were given to 32 (71%) and nutritional supplements to 7 (15·5%). A predominantly raw meat ration was being fed by 6 (13%) owners, 1 (2%) family fed a pasta-based ration and 1 (2%) dog was regularly given chocolate.

One (2%) dog was in an emaciated state, 5 (11%) were underweight, 28 (62%) were at ideal weight, 11 (24%) were overweight and no dogs were grossly obese.

Only 3 (7%) of the owners volunteered that their dog was drinking more, but when asked about water consumption 26 (58%) reported that their dog was drinking more frequently. Only 9 (20%) owners knew approximately how much their dog drank and 1 (2%) medium-sized dog was clinically polydipsic, -drinking more than 6 litres per day. Increased urinary frequency was reported for 11 (24%) dogs, 2 (4%) were exhibiting -inappropriate toileting by urinating in the house overnight, 1 (2%) had urinary incontinence and 1 (2%) male dog had stranguria.

Only 35 urine samples were available for analysis as the owners were unable to collect a sample or forgot and a sample could not be collected at the time of examination. Results of dip stick analyses are shown in Table 2. On refractometer examination of urine 1 (3%) dog had hyposthenuria (specific gravity SG<0·008) and 2 (6%) had isosthenuria (SG<1·010).

**Table 2:** Dip stick analysis results (n=35)

Dip stick test	Results (%)
No abnormality	12/35 (34)
Low specific gravity (<1·015)^*^	14/35 (40)
Protein present^*^	10/35 (29)
Leukocytes present^*^	5/35 (14)
Bilirubin present^*^	1/35 (3)
Glucose present	1/35 (3)
Ketones	0/35 (0)
Blood	0/35 (0)
Nitrite	0/35 (0)

Findings not reliable indicators of abnormality in dog urine collected by free catch However, positive results prompted further investigations to confirm if significant or not.

Most owners (39 (87%)) were able to describe the defaecation habits of their dogs and all were within an accepted range of frequency (1 to 3 times daily). Faeces was described as normal in form and appearance for 33 (85%) dogs but abnormalities were reported for 4 (10%) including the presence of mucus (2 cases), steatorrhoea (1 case), consistently soft stools (1 case) and faecal incontinence (1 case). The remaining 2 owners could describe frequency of defaecation but not the appearance of the faeces.

Otitis was present in 6 (13%) dogs with erythema and/or aural discharge with associated head shaking or ear scratching, 3 (7%) had epiphora and 1 (2%) had temporal muscle wastage.

Increased lens opacity (usually due to nuclear sclerosis but in four dogs due to cataract) was present in 29 (64%) dogs and pupillary light reflexes were slow in 3 (7%). Other abnormalities detected included conjunctivitis in 3 (7%) dogs, corneal ulceration in 1 (2%) and corneal oedema in 1 (2%).

Dental treatment was needed for 10 (22%) dogs, and 3 (7%) had severe halitosis due to their dental disease.

Abdominal pain was apparently present in 4 (9%) dogs with muscle tensing, vocalisation and turning towards the abdomen when palpated, and 1 dog with a palpable mass had acute pain, yelping and turning to bite the veterinarian. Four (9%) dogs had a pendulous abdomen and abdominal masses were palpable in 5 (11%). Prostatic enlargement was detected in 2 (22%) of entire males.

A cardiac murmur was present in 9 (20%) dogs, 5 (11%) had bradycardia (<60 beats per minute), 3 (7%) had an arrhythmia and 1 (2%) had tachycardia (>220 beats per minute). The dog with tachycardia was the same dog that had acute abdominal pain.

Panting throughout the consultation was a feature in 8 (18%) dogs, and abnormal respiratory noises (including wheezing, rhonchi, and inspiratory rales and crackles) were detected in 5 (11%). Coughing was reported for 4 (9%) dogs, 2 (4%) had tachypnoea and 1 (2%) had difficulty in breathing and cyanosis. The owner of the latter dog did not realise that the dog was in serious respiratory distress.

The results of examination of the musculoskeletal system are presented in Table 3, of examination of the dermatological system in Table 4, and the most important new problems identified in Table 5. Not all owners decided to have further diagnostic investigations or treatments, but a list of the procedures generated immediately following the screening consultations are -presented in Table 6. A summary of all 353 findings, an average of 7.8 per dog, is presented in Table 7.

**Table 3:** Findings of examination of the musculoskeletal system (n=45)

Finding	Number (%)
Reduced range of movement in joints	11/45 (24)
Pain	7/45 (15)
Stiffness	6/45 (13)
Crepitus	6/45 (13)
Muscle wastage	5/45 (11)
Reduced proprioception	4/45 (9)
Joint swelling	4/45 (9)
Weakness	3/45 (7)

**Table 4:** Findings of examination of the skin/subcutaneous tissue (n=45)

Finding	Number (%)
Masses suspicious for neoplasia (other)	8/45 (18)
Warts	5/45 (11)
Mammary tumours (n=25 females)	2/45 (4); 2/25 females (8)
Calluses	3/45 (7)
Lick granuloma	2/45 (4)
Alopecia	2/45 (4)

**Table 5:** Serious problems identified from screening elderly dogs (n=45) and which were not known to the practice

• Pain
• Severe respiratory distress
• Metastatic lung disease
• Abdominal masses
• Mammary neoplasia
• Liver disease
• Cardiac problems
• Rapid Weight loss
• Faecal incontinence
• Polydipsia/polyuria
• Osteoarthritis
• Steatorrhoea
• Dental work needed

**Table 6:** Further procedures generated as a result of screening

Diagnostic tests	Number (%)
Blood tests/pre-general anaesthetic bloods	16/45 (35%)
Radiography	10/45 (22%)
ECG	3/45 (7%)
Treatments	
Medical therapy	7/45 (15·6%)
Dental	10/45 (22%)
Surgery	2/45 (4%)
Euthanasia	2/45 (4%)

**Table 7:** Total number of problems identified from the screening of dogs over nine years of age (n=45). Most dogs had multiple problems

	Number of problems^1^ identified
History	
Owners history – mainly behavioural changes	67
Dietary history	8
Sub-total	75
Clinically significant findings	
Abnormal body weight	17
Abnormal thirst or urination	41
Urinalysis findings that prompted further investigations	31
Abnormal faeces/defaecation	4
Abnormal findings on examination of the head	10
Problems in the eyes – includes nuclear sclerosis	37
Abdominal examination	15
Dental examination	13
Cardiac examination	18
Respiratory examination	21
Musculoskeletal and neurological examination	46
Skin and subcutaneous tissues	25
Sub-total	278
Total	353

Problem defined as a finding that requires monitoring, a change in lifestyle, further diagnostic investigation or medical or surgical intervention

## DISCUSSION

There are several limitations of this study including the low number of dogs, involvement of only one practice and one veterinarian. The large number of problems detected raises concerns about possible bias in the recruitment method especially as the owners could have enrolled dogs for free screening knowing their dogs already had signs of illness. Follow-up contact with non-responsive owners could have increased recruitment numbers and reduced sampling bias. Blood screening may have identified additional problems, and the clinician may have missed some problems, especially in uncooperative animals or if owner histories were unreliable.

Veterinary nurses often run geriatric screening programmes in practice but, except for the history taking, the screening protocol used in this study could not be conducted by a veterinary nurse as it involved procedures that veterinarians are specifically trained to do. Furthermore, this protocol could be problematic for busy practices because of the time commitment required of a veterinary clinician.

In this study, at least 45 minutes was required for a full screen, which is far longer than practices usually allocate to a booster vaccination appointment, raising the question as to whether an adequate health check can be done at the time of vaccination.

There was an apparent over-representation of pedigree dogs compared to crossbreeds which probably did not reflect the demographic distribution of dogs in the practice but a breakdown of breeds in the practice was not available. The sex distribution of dogs probably did reflect the relative emphasis placed on neutering in the practice.

Owners often did not notice or recognise the importance of signs that were present in their dogs including increased thirst (initially reported to be present in 7% but actually present in 56% of dogs) and weight loss. No owners volunteered that their dog had lost weight, but all the dogs with a low body condition score, and some with ideal scores had lost weight in the period before the screening. Clinicians cannot rely on owners to observe and report important signs of age-related disease.

Increased sleeping was the most frequent owner observation, and altered sleep patterns are well recognised in humans with excessive daytime sleepiness and disturbed night time sleeping being reported in 50% of elderly people (Vitiello 2006). The underlying mechanisms are not fully understood and further studies are needed to determine the cause in dogs.

Gradual loss of sensory input and impairment of hearing and vision is to be expected due to ageing changes or age-related disease, e.g. nuclear sclerosis or cataracts in the lens of eyes. Nuclear sclerosis usually has minimal effects on vision whereas cataracts can progress to total blindness (Ofri 2008). Apparent reduced vision was reported by 20% of the owners but this did not correlate with ophthalmic disease except for the dogs with cataracts. This raises questions as to whether the clinician missed the presence of retinal or other disease, whether nuclear sclerosis itself may impair vision in some dogs and whether owner observations and perceptions of reduced vision are accurate.

As expected, many dogs showed musculoskeletal signs typical of an ageing population, particularly lameness or stiffness after exercise or a period of recumbency were reported in dogs with osteoarthritis and reports of “slowing down” were most often attributed to musculoskeletal disorders, but also to cardiorespiratory problems in some dogs.

The practice strongly advocated life-stage nutrition having a waiting room full of nutrition products so it was surprising that 89% of the owners did not feed a senior diet and many gave additional supplements, treats or snacks. It was concerning that a raw meat-based ration which can be both deficient in calcium, which could result in nutritional secondary hyperparathyroidism (Richardson and others 2000), and high in protein, the intake of which needs to be controlled in elderly animals with renal disease (Roudebush and others 2010), was being fed to 13% of the dogs. Also, one owner did not realise a pasta-based ration was inadequate and another that chocolate can be toxic. These findings highlight that owners do not always comply with nutritional advice given by practices.

Dip stick analysis of dog urine is of limited value as several parameters cannot be relied upon for accuracy (Archer 2005b). However, abnormal results do prompt further investigations and the finding of bilirubinuria was important in a diabetic dog which was found to have liver disease on follow-up serum biochemistry and bile acid stimulation tests.

Causes of continuous panting include stress, heat, pain or respiratory disease and further studies comparing a cohort of younger dogs are needed to determine if there is a significant difference in this sign with advancing age.

Owners knew about some problems before the screening consultation, but unrecognised problems were identified in 80% and over two-thirds of the dogs underwent further investigations or treatments which was greater than that reported by Hoskins (2004). This may be because of selection bias, or because relatively minor problems are reported in this study (e.g. nuclear sclerosis, warts).

The two dogs euthanased were a bright, apparently healthy collie bitch that had a small solitary inguinal mammary tumour and was scheduled for mastectomy but found to have lung metastases on screening radiography; and a faecally incontinent pointer. Whilst the latter owner knew the dog was very ill, the collie owner did not, and the outcome was unexpected. Thus screening can have a negative outcome for the animal and/or owner, and sometimes the risks of screening may outweigh the benefits. This was concluded by the Cochrane Database of Systematic Reviews when they looked at screening for breast cancer in humans (Nordic Cochrane Centre 2012).

Further studies are needed to confirm whether the results of this study could be replicated in other practices, and a longer follow-up period would be useful to measure ultimate outcomes and to quantify the economic consequences of running such a programme.

### MAIN CONCLUSIONS AND CLINICAL -SIGNIFICANCE

In this cohort of 45 dogs aged over nine years of age attending a first opinion veterinary practice screening identified unrecognised and unreported signs of age-related disease and the presence of risk factors resulting in recommendations for lifestyle changes (e.g. diet), ongoing monitoring, further diagnostic investigations, and medical and surgical interventions. A mean of 7·8 problems were identified per dog and previously unrecognised problems were found in 80% of the dogs. Over two-thirds of the dogs underwent further investigative procedures or interventions and in some cases unrecognised life-threatening problems were found. Signs of pain were not recognised by many owners yet approximately one in four dogs required analgesia. Clinicians cannot rely on owners to report common signs of age-related -diseases such as increased thirst, weight loss, or pain and should consider running screening clinics for elderly patients and educate clients in recognition of important signs.

### ETHICS

All data from this study was collected, stored and used anonymously in accordance with the Data Protection Act 1998.

## References

[b1] American Animal Hospital Association (2005). AAHA senior care guidelines for dogs and cats. Journal of American Animal Hospital Association.

[b2] Alef M, von Praun F, Oechtering G (2008). Is routine pre-anaesthetic haematological and biochemical screening justified in dogs?. Veterinary Anaesthesia and Analgesia.

[b3] Archer J, Villiers E, Blackwood L (2005a). Interpretation of laboratory data. Chapter 2. BSAVA Manual of Canine and Feline Clinical Pathology.

[b4] Archer J, Villiers E, Blackwood L (2005b). Urine analysis. Chapter 10. Manual of Canine and Feline Clinical Pathology.

[b5] Davies M (1996). Canine and Feline Geriatrics.

[b6] Davies M (2011). Internet users’ perception of signs commonly seen in old animals with age-related diseases. Veterinary Record.

[b7] Fort Dodge Index (FDI) (2010).

[b8] Galena HJ (1992). Complications occurring from diagnostic venipuncture. Journal of Family Practice.

[b9] Hoskins JD (2004). Geriatrics and Gerontology of the Dog and Cat.

[b10] Joubert KE (2007). Pre-anaesthetic screening of geriatric dogs. Journal of the South African Veterinary Association.

[b11] Nordic Cochrane Centre (2012). www.cochrane.dk/screening/index-en.htm.

[b12] Ofri R (2008). Lens. Chapter 13. Slatter's Fundamentals of Veterinary Ophthalmology.

[b13] Richardson DC, Zentek J, Hazewinkle HAW, Toll PW, Zicker SC, Hand Thatcher (2000). Remillard and Roudebush. Developmental orthopaedic disease of dogs. Small Animal Clinical Nutrition.

[b14] Roudebush P, Polzin DJ, Adams LG, Towell TL, Forrester SD (2010). An evidence-based review of therapies for canine chronic kidney disease. Journal of Small Animal Practice.

[b15] Vitiello MV (2006). Sleep in normal aging. Sleep Medicine Clinics.

